# Scrub typhus in two COVID-19 patients: a diagnostic dilemma

**DOI:** 10.2217/fmb-2021-0163

**Published:** 2022-01-19

**Authors:** Chandan Kumar Thakur, Priyam Batra, EV Vinayaraj, K Sreenath, Nisha Rathor, Urvashi B Singh, Ridhima Bhatia, Ajisha Aravindan, Naveet Wig, Randeep Guleria, Rama Chaudhry

**Affiliations:** 1 Department of Microbiology, All India Institute of Medical Sciences, Ansari Nagar, New Delhi, 110029, India; 2 Department of Anesthesiology, Pain Medicine & Critical Care, All India Institute of Medical Sciences, Ansari Nagar, New Delhi, 110029, India; 3 Department of Medicine, All India Institute of Medical Sciences, Ansari Nagar, New Delhi, 110029, India; 4 Department of Pulmonary, Critical Care & Sleep Medicine, All India Institute of Medical Sciences, Ansari Nagar, New Delhi, 110029, India

**Keywords:** acute febrile illness, co-infection, COVID-19, diagnosis, scrub typhus

## Abstract

The authors describe a case series of co-infection with COVID-19 and scrub typhus in two Indian patients. Clinical features like fever, cough, dyspnea and altered sensorium were common in both patients. Case 1 had lymphopenia, elevated IL-6 and history of hypertension, while case 2 had leukocytosis and an increased liver enzymes. Both patients had hypoalbuminemia and required admission to the intensive care unit; one of them succumbed to acute respiratory distress syndrome further complicated by multiple organ dysfunction syndrome. Seasonal tropical infections in COVID-19 patients in endemic settings may lead to significant morbidity and mortality. Therefore, high clinical suspicion and an early diagnosis for co-infections among COVID-19 patients are essential for better patient management.

The ongoing COVID-19 pandemic, which began in December 2019, has posed a serious public health threat globally due to its varying presentations as well as the difficulty in diagnosis and treatment [1]. COVID-19 has an incubation period of 2–14 days with varying manifestations ranging from asymptomatic to mild fever to severe, fatal pneumonia [2]. Various tropical diseases such as scrub typhus and leptospirosis also have such varied presentations and may present with acute respiratory distress syndrome (ARDS) and multiorgan dysfunction syndrome (MODS), which can mimic the current COVID-19 pandemic in endemic countries such as India. Clinicians must be aware of the presence of scrub typhus in endemic regions especially in the monsoons and post-monsoon season. If diagnosed early, prompt treatment can be initiated for the patient [3]. Here, the authors would like to describe two cases of co-infection with SARS-COV-2 and scrub typhus. This article further attempts to express an opinion on the need to address and timely diagnose cases of co-infection with scrub typhus and COVID-19, especially in endemic areas.

## Case presentation

In August 2020, a 55-year-old hypertensive male patient, a resident of Madhya Pradesh (a state in central India), presented to the emergency department with fever, cough, dyspnea and altered sensorium. The patient was a known case of seizure disorder and was diagnosed to have meningoencephalitis and COVID-19 lower respiratory tract infection by cartridge-based nucleic acid amplification test (CBNAAT) (Cepheid's GeneXpert) in the emergency department. He was admitted to the intensive care unit (ICU) and was started on ceftriaxone, vancomycin and doxycycline, and his anti-hypertensive drugs were continued. Ceftriaxone and vancomycin were added as empiric antibiotic therapy for meningoencephalitis, and doxycycline was added to take care of other pathogens causing atypical pneumonia. The patient had lymphopenia (lymphocyte count: 9%; reference range: 20–40%), decreased albumin level (3 gm/dl; reference range: 3.2–4.8 gm/dl) and increased IL-6 (27.26; reference range: 5–15 pg/ml) at the time of admission. He responded well to the treatment and was shifted out of the ICU on day 6. He was successfully discharged after 21 days of admission.

In June 2020, a 35-year-old male, a resident of Delhi (a state in north central India) with no prior comorbidities was brought to the emergency department in altered sensorium with fever, cough and dyspnea for the past week. His laboratory investigations revealed increased liver enzymes (alanine transaminase [ALT]; 95 u/l; reference range: 5–45 u/l; aspartate transaminase [AST]; 51 u/l; reference range: 5–40 u/l; and alkaline phosphatase [ALP]; 587 u/l; reference range: 80–240 u/l) and decreased albumin level (2.8 gm/dl; reference range: 3.2–4.8 gm/dl). The patient had ARDS and was found to be COVID-19 positive by real-time reverse transcription polymerase chain reaction (RT-PCR) [4] in the authors' institute. The patient was mechanically ventilated in the emergency department and was started on hydroxychloroquine, piperacillin/tazobactam and doxycycline. Hydroxychloroquine was started for COVID management; doxycycline and piperacillin/tazobactam were given empirically for other ARDS-causing atypical pathogens. However, his condition kept deteriorating and on day 6 he was shifted to the ICU, where piperacillin/tazobactam was escalated to meropenem. However, the patient did not respond to treatment and went into multiorgan failure and finally succumbed to the infection on day 14. Detailed clinical features and laboratory investigations of both patients are tabulated in Tables 1 & 2.

**Table 1.** Baseline information for the two patients.

Clinical features	Case 1	Case 2
Age (years)	55	35
Sex	Male	Male
Fever	Present	Present
Cough	Present	Present
Dyspnea	Present	Present
Myalgia	Absent	Absent
Vomiting	Absent	Absent
Diarrhea	Absent	Absent
Pulse rate (per min)	54	140
Respiratory rate (per min)	20	38
Blood pressure (mmHg)	100/58	130/90
Oxygen saturation (room air)	100%	50%
Altered sensorium	Present	Present
Duration of hospital stay (days)	21	14
**Comorbidities**		
Diabetes	No	No
Hypertension	Yes	No
Bronchial asthma	No	No
COPD	No	No
Immunocompromised	No	No
Cardiovascular diseases	No	No
Renal diseases	No	No
TB	No	No
Malignancy	No	No

COPD: Chronic obstructive pulmonary disease; TB: Tuberculosis.

**Table 2.** Laboratory parameters of the two patients.

Laboratory parameters	Reference range	Case 1	Case 2
Hemoglobin	Female: 11–15 g/dl; male: 13–17 g/dl	11.2	11.1
Red blood cell count	3.8–4.8 (10^6^/μl)	3.65	3.25
White blood cell count	4–11 (10^3^/μl)	11.88	16.38
Neutrophils	40–80 (%)	82.5	ND
Lymphocytes	20–40 (%)	9.9	ND
Monocytes	2–10 (%)	7	ND
Eosinophils	1–6 (%)	0.2	ND
Basophils	1–2 (%)	0.4	ND
Platelet count	150–400 (10^3^/μl)	262	277
Total bilirubin	0.3–1.2 mg/dl	0.4	0.7
ALT	5–45 u/l	24	95
AST	5-40 u/l	34	51
ALP	80–240 u/l	65	587
Urea	<50 mg/dl	19	53
Creatinine	0.5–1.1 mg/dl	0.6	0.6
Sodium	132–146 mmol/l	138	137
Potassium	3.5–5.5 mmol/l	4	5.2
Total protein	5.7–8.2 g/dl	6	5
Albumin	3.2–4.8 g/dl	3	2.8
Globulin	2.5–3.4 g/dl	2.9	2.2
LDH	140-280 u/l	304	ND
IL-6	5-15 pg/ml	27.26	ND
Serum ferritin	10–291 ng/ml	220.5	ND
**Co-infections**			
Scrub typhus IgM ELISA (OD)	≥0.89	1.2	1.1
Scrub typhus IgM IFA	≥64	+	–
Nested PCR (56 kDa)	483 bp	–	–
Serology for *Rickettsia* spp.*, Leptospira* spp.*, Mycoplasma pneumoniae*, *Chlamydia pneumoniae* and *Legionella pneumophila*	Refer to Table 3	–	–
Molecular tests for *Rickettsia* spp., *Leptospira* spp., *M. pneumoniae* and *L. pneumophila*	Refer to Table 3	–	–

ALT: Alanine transaminase; ALP: Alkaline phosphatase; AST: Aspartate transaminase; bp: Basepair; ELISA: Enzyme-linked immunosorbent assay; ESR: Erythrocyte sedimentation rate; IFA: Indirect immunofluorescence assay; kDa: Kilodalton; LDH: Lactate dehydrogenase; ND: Not done; OD: Optical density.

Screening for other diseases responsible for febrile illness such as leptospirosis, atypical pneumonia (*Mycoplasma pneumoniae, Chlamydia pneumoniae* and *Legionella pneumophila*), rickettsioses (spotted fever and typhus group) and scrub typhus were also performed to rule out other differentials, which are endemic in India. Both patients were serologically tested positive for scrub typhus using IgM enzyme-linked immunosorbent assay (ELISA) (InBios, WA, USA), whereas indirect immunofluorescence assay (IgM IFA) (Fuller Laboratories, CA, USA) for scrub typhus was only positive in case 1. The predetermined cut-offs for ELISA and IFA for scrub typhus were used [5]. However, serological tests for other organisms remained negative (details of tests for other organisms are given in Table 3).

**Table 3.** Serological and molecular tests used to rule out other pathogens.

Organism	Serology	Molecular targets, test performed	Ref.
Rickettsia spotted fever and typhus group	IgM IFA (Fuller Laboratories, CA, USA) cut-off 1:64	*gltA* gene, real-time PCR	[26]
*Leptospira* spp.	IgM ELISA (Panbio Pty., Queensland, Australia) cut-off >11	*16s rRNA*, conventional PCR	[27]
**Atypical bacteria** *Mycoplasma pneumoniae*	IgM ELISA (NovaTec Immundiagnostica GmbH, Dietzenbach, Germany) cut-off >11	*CARDS toxin* gene, real-time PCR	[28]
*Chlamydia pneumoniae*	IgM ELISA (NovaTec Immundiagnostica GmbH, Dietzenbach, Germany) cut-off >11	Not done	
*Legionella pneumophila*	IgM ELISA (NovaTec Immundiagnostica GmbH, Dietzenbach, Germany) cut off >11	*ssrA* gene, real-time PCR	[29]

ELISA: Enzyme-linked immunosorbent assay; IFA: Indirect immunofluorescence assay.

DNA was extracted from EDTA-anticoagulated whole blood samples of patients with positive serology. Nested PCR assay targeting the 56 kDa type-specific antigen (*tsa*) gene of scrub typhus was performed [6]. Additionally, these samples were screened for other tropical pathogens, including *Rickettsia*, *Leptospira* and atypical bacteria (Table 3). Molecular detection for *Leptospira* spp. by PCR and *Mycoplasma pneumoniae* and *Legionella pneumophila* by real-time PCR were found to be negative. Molecular tests for *Chlamydia pneumoniae* could not be performed. However, nested PCR for *Orientia tsutsugamushi* and real-time PCR for *Rickettsia* spp. were also negative for both cases.

## Discussion

Scrub typhus is a mite-borne bacterial infection caused by the obligate intracellular organism *Orientia tsutsugamushi*. Annually, one million cases are estimated to occur globally and approximately one billion people are at risk with substantial mortality rates [7]. It is endemic in almost every state of India and accounts for up to 15–30% of all febrile episodes [8]. India is the second most infected country with COVID-19 in the world after the USA [9]. Scrub typhus in India occurs mostly during the rainy season (July to November) and is characterized by myriad clinical manifestations such as fever, headache, rash, myalgia, dyspnea, lymphadenopathy, ARDS and MODS [3]. These symptoms can mimic the clinical symptoms of an ongoing outbreak of COVID-19 in the absence of characteristic eschar. Although the presence of eschar is highly suggestive of scrub typhus, looking for it in COVID-19 patients is difficult, as they require physical separation. Moreover, scrub typhus had not been suspected in these patients; hence, a detailed physical examination was not undertaken by overburdened clinicians in the present epidemic.

Blacksell *et al*. have described the modified scrub typhus infection criteria (mSTIC) for laboratory diagnosis of scrub typhus, which includes qPCR using the *Orientia* spp. 47 kDa *htra* gene, a single admission IgM IFA titer  ≥1:3200, IFA ≥ 1:3200 on admission or fourfold rise to ≥3200 and a combination of PCR and IFA positivity [10]. However, PCR and IFA are expensive and not easily available in the majority of Indian hospitals. Also, the high cost of IFA restricts the determination of end point titers in every positive case. Previous Indian studies have highlighted the fact that while a fourfold increase in antibody titers in paired sera is expected for diagnosis, a prompt diagnosis at the time of admission is required to guide initial treatment. Furthermore, the availability of convalescent sera is limited, as most cases do not come for follow-ups. Hence, following such diagnostic algorithm may not be feasible in the majority of the tertiary care referral hospitals located in India [5,11]. A study from Thailand by Blacksell *et al.* found that an admission diagnosis of scrub typhus at a cut-off OD of 0.5 using ST InBios IgM ELISA corresponds to an IFA reciprocal titer cut-off of ≥1600. The use of 0.5 as the cut-off OD in ELISA was demonstrated to have sensitivity and specificity of 93% and 91%, respectively, and may thus be used as an excellent alternative to the gold standard IFA[12]. Another study from Bangladesh by Blacksell *et al*. concluded that cut-off OD within the range of 0.75–1.25 using InBios ST IgM ELISA was the most suitable cut-off OD for the diagnosis of scrub typhus [10]. In the present study, IgM antibodies against scrub typhus were detected using ELISA (cut-off >0.89) in both patients with OD more than 1.00. However, IFA was positive in only one case. IFA is considered a reference test for the diagnosis of scrub typhus, but commercial IFA slides are coated with only four serotypes: Karp, Kato, Gilliam and Boryong. However, diverse serotypes of *Orientia tsutsugamushi*, which may be missed by IFA, are circulating in the world [13]. A molecular assay such as PCR is usually positive in the first week of disease during rickettsemia. It has low sensitivity beyond the first week of illness [14]. The PCR negativity in these two patients might be attributed to the late course of scrub typhus and the use of antibiotics such as doxycycline during COVID-19 therapy. In addition, it is recommended that a buffy coat be used due to high sensitivity rather than whole blood for scrub typhus PCR, which might have affected the PCR positivity [15]. The limitation of this study is that the authors were not able to demonstrate a rising antibody titer because of the unavailability of convalescent serum. In addition, they were unable to determine whether the death of one patient in this report was attributed to co-infection or COVID-19 alone, because complications like ARDS and MODS are associated with scrub typhus as well COVID-19.

Scrub typhus is grossly under-reported and underdiagnosed, attributable to the misperception that this disease is only a concern in vigorously forested zones. The clinical presentation often mimics that of other common febrile illnesses that share similar seasonal patterns, such as chikungunya and dengue virus infection, creating a diagnostic dilemma and delay in definitive therapy that may lead to adverse clinical outcomes. Thus, the possibility of the co-existence of scrub typhus with COVID-19 during monsoon and post-monsoon season in endemic settings cannot be ruled out; therefore, it should be looked at cautiously and treated appropriately. Previously, the authors reported mortality in five patients due to scrub typhus during the chikungunya outbreak in the absence of timely suspicion, diagnosis and treatment [16]. A recent case report from Thailand stated that COVID-19 can present as an acute febrile illness and can be difficult to distinguish from other tropical diseases, particularly when respiratory symptoms are absent [17].

Recent studies have described the co-infection of COVID-19 with other respiratory pathogens like influenza, *Mycoplasma pneumoniae, Chlamydia pneumoniae, Legionella pneumophila, Klebsiella pneumoniae, Acinetobacter baumanii, Aspergillus fumigatus* [18–23] and *Candida auris* [24]; however, information related to co-infection of COVID-19 with scrub typhus is lacking. Bacterial co-infections in the presence of viral pneumonia have been shown to be a major cause of morbidity and mortality [25]. Hence, there is a great necessity to include scrub typhus in the differential diagnosis of patients presenting with fever in tropical areas, especially with ARDS and MODS, during the current COVID-19 pandemic.

## Conclusion

Scrub typhus is a prominent cause of acute febrile illness in the Asia–Pacific region. The most essential management challenge is the institution of appropriate therapy in a timely and effective manner. During outbreaks of viral fever, scrub typhus cases may get missed due to overlapping clinical presentations. Co-infection with such seasonal tropical infections in endemic settings leads to significant morbidity and mortality. Therefore, high clinical suspicion for co-infections among COVID-19 patients during an early stage of illness and precise diagnosis are essential for better patient management.

**Summary points**

- The authors describe a case report of co-infection with COVID-19 and scrub typhus in two Indian patients.
- Tropical diseases such as scrub typhus also have varied presentations and may present with acute respiratory distress syndrome and multiorgan dysfunction syndrome, which can mimic the current COVID-19 in endemic countries such as India.
- The possibility of the co-existence of scrub typhus with COVID-19 during monsoon and post-monsoon season in endemic settings cannot be ruled out, and therefore it should be looked at cautiously and treated appropriately.
- Bacterial co-infections in the presence of viral pneumonia can result in morbidity and mortality.
- Clinicians must be aware of the existence of scrub typhus with or without COVID-19 in endemic areas, particularly in the absence of eschar.
